# Algal polysaccharides: new perspectives for the treatment of basal ganglia neurodegenerative diseases

**DOI:** 10.3389/fnana.2024.1465421

**Published:** 2024-10-16

**Authors:** Alessandra Marinho Miranda Lucena, Eudes Euler de Souza Lucena, Sebastião Pacheco Duque Neto, Leonardo Thiago Duarte Barreto Nobre, Hugo Alexandre Oliveira Rocha, Rafael Barros Gomes Câmara

**Affiliations:** ^1^Graduate Program in Biochemistry and Molecular Biology – PPgBBM, Bioscience Center, Federal University of Rio Grande do Norte, Natal, Brazil; ^2^Multicampi School of Medical Sciences of Rio Grande do Norte, Federal University of Rio Grande do Norte, Caicó, Brazil

**Keywords:** polysaccharides, algae, neuroprotection, neurodegenerative, basal ganglia

## Abstract

The objective of this review was to verify the therapeutic effect of polysaccharides derived from algae in neurodegenerative disease models involving the basal ganglia. To achieve this goal, a literature search was conducted in PubMed, Science Direct, Scopus, Web of Science, Embase, and Google Scholar databases. The descriptors “neuroprotective or neural regenerative or immunomodulatory activity or neuroprotection,” “polysaccharide or carbohydrate or carbohydrate polymers,” “marine algae or seaweed,” and “basal ganglia” according to the Preferred Reporting Items for Systematic reviews and Meta-Analyses (PRISMA) methodology were used. This methodology involved the steps of searching, pre-selection, and inclusion of articles. A total of 737 records were identified. Following the data analysis, 698 studies were excluded, resulting in a final sample of 8 studies. Species such as *Turbinaria decurrens*, *Gracilaria cornea*, *Chlorella pyrenoidosa*, *Arthrospira (Spirulina) platensis*, *Fucus vesiculosus*, and *Laminaria japonica* have demonstrated significant neuroprotective effects. This review suggests that polysaccharides derived from marine algae possess therapeutic potential for neuroprotection, modulation of inflammation, and amelioration of functional deficits. Their use in neurodegenerative disease models warrants further consideration.

## Introduction

Basal ganglia (BG) or *basal nucleus* as formally named by International Neuroanatomical Terminology (INT) (FIPAT, 2017) are complex neuroanatomical and hodological subcortical structures involved in both motor and “non-motor” functions. The neuroanatomical topography of the basal nucleus is composed of (1) the striatum (subdivided into the caudate nucleus, putamen, and nucleus accumbens), (2) the globus pallidus lateral, (3) the globus pallidus medial, (4) the subthalamic nucleus, and (5) the substantia nigra (Rocha et al., 2023).

The functional organization of basal nuclei circuitry includes dopaminergic, GABAergic, and glutamatergic intrinsic and extrinsic projections (afferent and efferent), in both direct and indirect pathways (Simonyan, 2019). The three most important dopaminergic mesencephalic ascendent systems are the nigrostriatal, mesolimbic, and mesocortical or mesocorticolimbic pathways. Dysfunction in these pathways can lead to conditions such as Parkinson’s disease (PD) and Huntington’s disease (HD) (Andres and Darbin, 2018; Obeso et al., 2014).

In HD, early pathological events such as an increase in the production and release of inflammatory mediators (e.g., IL-6, IL-8, and TNF-ɑ) can be observed (Jia et al., 2022). In PD, oxidative stress following mitochondrial damage due to environmental risk factors or chronic microglial inflammation mediates the activation of ɑ-synuclein-rich protein aggregates in the form of Lewy bodies, which are also present in the autosomal dominant form of PD (Vázquez-Vélez and Zoghbi, 2021). The overexpression of ɑ-synuclein, in turn, leads to mitochondrial death (Vázquez-Vélez and Zoghbi, 2021). Excitotoxicity, which is also related to oxidative stress, causes several intracellular damages in microglia, leading to the constant expression of pro-inflammatory cytokines, morphological changes, and cell death (Jia et al., 2022).

Neurodegeneration is a multifaceted process that leads to neuronal dysfunction. It involves intricate intracellular and extracellular mechanisms operating through diverse biochemical pathways. When considering effective prevention and therapy for neurodegenerative diseases, it is essential to consider all of these interconnected factors. Several studies have shown that neuroprotective agents from natural sources play an important role in the prevention and treatment of neurodegenerative diseases (NDDs). Among the molecules studied, polysaccharides extracted from plants, fungi, and algae are noteworthy (Dhahri et al., 2021; Wang et al., 2022). Natural polysaccharides that are capable of preventing or interfering with the initial neurodegenerative processes hold promise for preventive interventions.

Due to their multiple biological activities, especially antioxidant and anti-inflammatory properties, and their low risk of toxicity and adverse effects (Xu et al., 2022), polysaccharides have great potential for the prevention and treatment of NDD. Additionally, these molecules could reverse neurodegeneration and improve cognitive function, learning, memory, and motor skills, which could be valuable for therapeutic purposes (Dhahri et al., 2021).

Notably, algae have emerged as a rich source of novel chemical compounds with significant biological effects, having been used for nutritional and medicinal purposes for many years (Silva et al., 2023). The cultivation and consumption of marine macroalgae are expanding widely and proving to be safe and sustainable in several aspects, enabling the biotechnological development of economically responsible therapeutic alternatives (Sugumaran et al., 2022).

Therefore, this study aims to systematically review original articles that investigated the use of polysaccharides from marine algae in the treatment of neural damage and diseases associated with the basal ganglia.

## Methods

The present study comprises a systematic review of the literature in order to gather and analyze studies concerning the effects of polysaccharides from marine algae on experimental animal models of neuroprotection and neuroregenerative aspects involving the basal ganglia. The approach was developed in accordance with well-defined stages, including search, identification, selection, and eligibility strategies. For database searches, we used specific terminologies, filters, and descriptors relevant to articles published in six databases, namely PubMed, Science Direct, Scopus, Web of Science, Embase, and Google Scholar.

**Figure 1:**
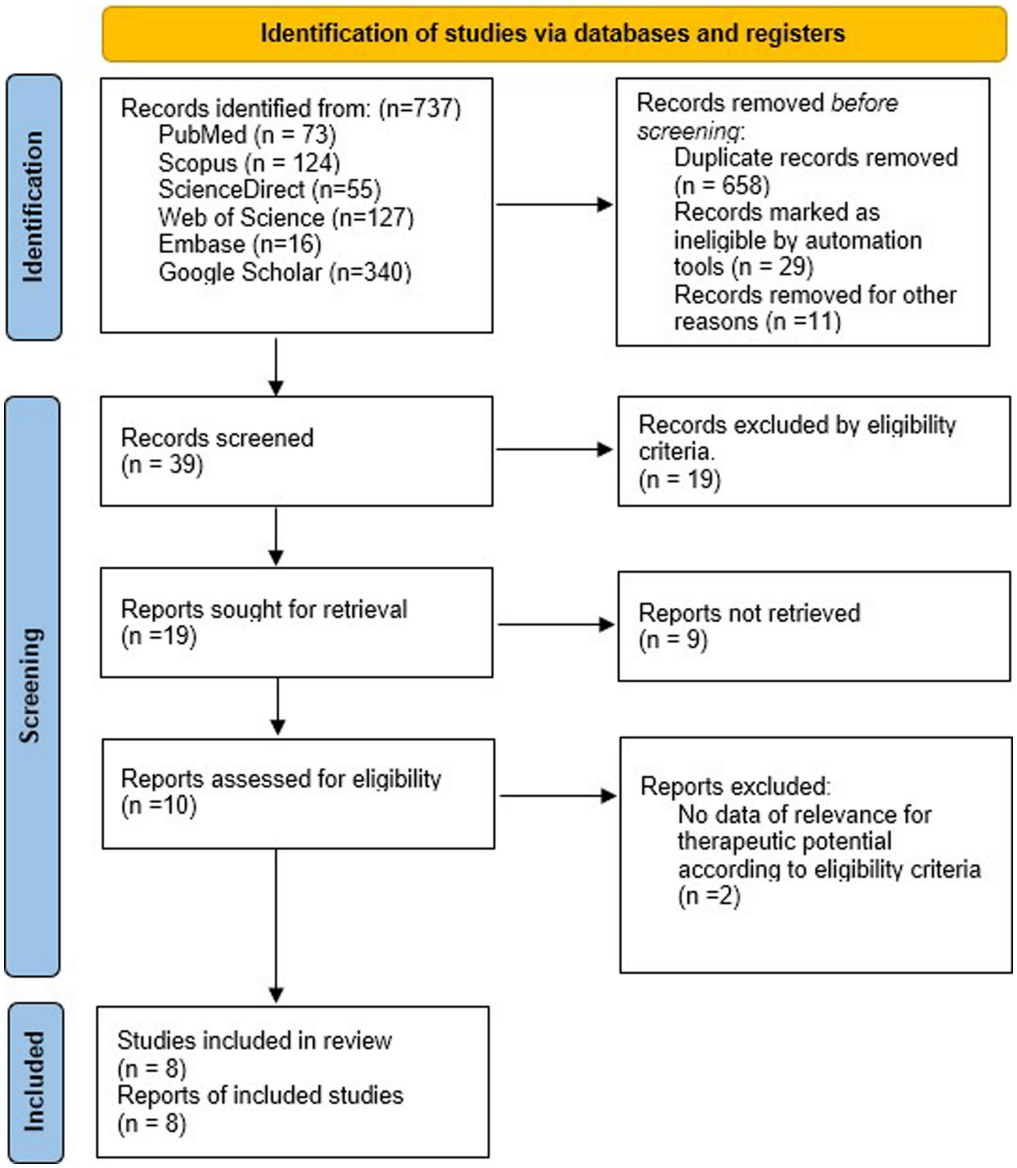
Synthesis of the search process according to the PRISMA model.

The selected terms were based on the technical vocabulary commonly used to index articles in the field of health sciences. These terms align with the Medical Subject Headings (MeSH) provided by the US National Library of Medicine (NLM) for accurate descriptor-based searching: “neuroprotective or neural regenerative or immunomodulatory activity or anti-inflammatory or neuroprotection” and “polysaccharide or carbohydrate or carbohydrate polymers” and “marine algae or seaweed” and “Basal ganglia” or “Basal nuclei” or “striatum” or “caudate nucleus” or “putamen” or “nucleus accumbens” or “globus pallidus” or “subthalamic nucleus” or “substantia nigra.” The combinations of these keywords were used to search for all studies that analyzed the effects of polysaccharides from marine algae on experimental models of both neurological disorders and NDD. This review was conducted following the recommendations of the Preferred Reporting Items for Systematic reviews and Meta-Analyses (PRISMA) (Page et al., 2021). There were no disagreements regarding the selection of the sample, and there were no temporal limitations. Figure 1 shows the search process.

In our study, two expert evaluators independently conducted a systematic search for relevant articles. They screened the titles and abstracts and resolved any discrepancies through subsequent consensus meetings. Articles were limited to those published in English. After initial screening, full-length publications were read and compared. We included experimental studies that examined the effects of polysaccharides *in vivo*, focusing on specific pathological conditions. Additionally, we considered studies that assessed behavioral improvements related to the pathology. The exclusion criteria encompassed non-original articles, studies using cell culture models, analyses combining neural effects with other compounds (without an isolated algae group), and the absence of a comparable control group.

In this systematic review, we have established rigorous eligibility criteria for the inclusion of studies, including experiments conducted on murine or rat models with induced neurological lesions, treatment administered either before or after the occurrence of damage or intervention, comprehensive analysis of the effects on injury and underlying action mechanisms, and the inclusion of animal models exhibiting clinical symptoms relevant to the specific pathologies under investigation. Additionally, a questionnaire was used to assess how well a manuscript complies with the ARRIVE Essential 10 (Percie du Sert et al., 2020). This questionnaire was utilized to describe comparative experiments conducted on living animals, considering the following aspects: study design, sample size, inclusion and exclusion criteria, randomization, blinding, outcomes measures, statistical methods, experimental animals, experimental procedures, and results.

However, the evaluated studies exhibited notable inconsistencies: the inducing agents used to simulate neurodegenerative diseases varied across studies, treatment durations were heterogeneous, and behavioral tests employed diverse methodologies. Neuronal damage simulation utilized different compounds, each acting through distinct mechanisms. Furthermore, these compounds were administered at different doses via different routes, further contributing to the study heterogeneity.

## Results

In this study, researchers initially collected 737 records. After rigorous data analysis, 698 studies were excluded based on specific criteria. Ultimately, eight studies fulfilled the inclusion and quality assessment criteria. Figure 1 shows the research flow. The selected articles underwent thorough examination, focusing on authorship, publication year, algae species, research methods, evaluation, and key findings (Table 1).

**Table 1:** Summary of selected studies in this review.

Author and year	Algae/class	Objectives/study model	Evaluation	Effect/treatment mechanisms
Meenakshi et al. (2016)	*Turbinaria decurrens* (Pheophyceae)	This study aimed to investigate the neuroprotective effect of fucoidan against dopaminergic neurodegeneration. Male mice *Mus musculus* (C57BL/6) ranging 25–30 g were randomized and divided into four experimental groups: saline (control), 1-methyl-4-phenyl-1,2,3,6-tetrahydropyridine (MPTP), MPTP + fucoidan from *T. decurrens* and fucoidan alone. These substances were injected intraperitoneally.	Behavioral tests (including open-field test, narrow beam walking test, hang test, and rotarod test) were performed. Dopamine and its metabolites were quantified; Estimation of non-enzymatic (lipid peroxidation products and reduced glutathione) and enzymatic antioxidants (superoxide dismutase, catalase, glutathione peroxidase), Western blotting analysis (tyrosine hydroxylase), and immunohistochemistry (tyrosine hydroxylase, dopamine transporter [DAT], ß-actin) were performed.	The pretreatment of fucoidan in MPTP-administered mice resulted in increased peripheral and central movements; the mice also showed a reduced time to cross the beam and a decrease in the frequency of foot slip errors. Additionally, they were able to withstand the rotarod for a considerable amount of time and showed a significant increase in the hanging time. Antioxidants and dopamine levels were increased in the fucoidan-treated mice when compared to MPTP mice. In immunohistochemistry, the increase of tyrosine hydroxylase (TH)-positive cells in the fucoidan-treated group is correlated with the TH protein levels in the substantia nigra and corpus striatum. In immunoblotting, expression of TH and DAT antibodies in MPTP mice were reduced and reversed in other groups.
Souza et al. (2017)	*Gracilaria cornea* (Florideophyceae)	This study aimed to evaluate the neuroprotective effects of sulfated polysaccharide (SA-Gc) in a Parkinson’s disease (PD) rat model induced by 6-hydroxydopamine (6-OHDA). The PD model in male rats was induced by an intrastriatal injection of 6-OHDA, followed by a single administration of SA-Gc.	After 14 days, the animals were screened for behavioral impairment using open-field, rotarod, and apomorphine-induced rotation tests. The striatum (ipsilateral and contralateral), the hippocampus, and the prefrontal cortex were dissected and used for neurochemical (noradrenaline—NE; dopamine—DA; 3,4 dihydroxyphenylacetic acid—DOPAC; homovanillic acid—HVA and serotonin—5-HT), and transcriptional analyses (Brain-derived neurotrophic factor—BDNF; Nuclear factor kappa Bp65subunit—p65; Inducible nitric oxide synthase—iNOS; Interleukin-1b—IL-1; b-Actin—b-Act; a-Tubulin—a-Tub; Glyceraldehyde-3 -phosphate-dehydrogenase—GAPDH).	In the behavioral tests analyzed, SA-Gc restored the locomotor performance and grooming to basal conditions in animals subjected to 6-OHDA, along with a marked increase in the rearing frequency to reach 1.9 times. The latency to fall was prolonged in the rotarod performance test to approximately normal values, signifying enhanced motor coordination and balance. In addition, SA-Gc showed transcriptional modulation of the NFkB/IL-1b pathway, NFkB/iNOS/NO2 and NO3 axis, and subsequent downstream vicious inflammatory and oxidative cascades that promote damage to dopaminergic neurons.
Zhang et al. (2014)	*Laminaria japonica* (Laminariaceae)	This study aimed to assess the effects of fucoidan on the neurochemical loss of nigral DA neurons and behavioral deficits in a PD rat model induced by 6-OHDA. The PD model in male rats was induced by an intrastriatal injection. These PD rats were randomly divided into three groups: (i) 6 OHDA rats treated with intraperitoneal (i.p.) injection of saline once daily for 3 weeks, (ii) 6-OHDA rats treated with fucoidan (10 mg/kg, i.p.) once daily for 3 weeks, and (iii) 6-OHDA rats treated with fucoidan (20 mg/kg, i.p.) once daily for 3 weeks.	To examine rotational behavior induced by apomorphine (APO), rats were placed in a cylinder rotameter. Locomotor activity was assessed in Truscan-automated activity chambers connected to a digiscan analyzer. Sections through the striatum and substantia nigra (SN) were collected. TH, neuronal nuclei (NeuN), glial fibrillary acidic protein (GFAP), CD11B integrin, and NADPH oxidase 1 (Nox1) immunoreactive protein were examined. TH or b-actin or Nox1 protein concentrations were determined through the Western blot analysis.	Chronic fucoidan administration mitigated the motor dysfunction induced by 6-OHDA. At a dose of 20 mg/kg, fucoidan significantly reduced rotational activities. A longer period (3 weeks) of treatment with fucoidan showed a significant effect at doses 10 mg/kg and 20 mg/kg. A significantly smaller decrease in locomotor activity was seen in rats treated with fucoidan. Similarly, fucoidan reduced the loss of DA neurons in the substantia nigra pars compacta (SNc) and DA fibers in the striatum in 6 OHDA-lesioned rats. Moreover, it was found that fucoidan inhibited the 6-OHDA-stimulating expression of Nox1 in both tyrosine hydroxylase (TH)-positive neurons and non-TH-positive neurons, preventing Nox1-sensitive oxidative stress and cell damage in SNc neurons. Fucoidan also effectively inhibited nigral microglial activation.
Chen et al. (2014)	*Chlorella pyrenoidosa* (Trebouxiophyceae)	The aim of this study was to investigate the effects of polysaccharides *Chlorella pyrenoidosa* (CPS) on motor activity, dopamine expressions, microglial activation, and peripheral immunomodulatory responses in MPTP-induced mouse model of PD. Male C57BL/6 mice were randomly divided into treatment and control groups. Treatment groups received CPS while control, negative, and positive groups were administered water. Experimental parkinsonism was established by intraperitoneal injections of MPTP on the 11th day 1 h after oral administration of CPS or water. The positive group was treated with MPTP followed by a daily dose of L-dopa with benserazide from the 12th day.	Behavioral tests (pole test and gait test) were performed; DA and its metabolites were quantified, DOPAC and HVA were carried out; Ribonucleic Acid (RNA) isolation, cDNA synthesis, and quantitative real-time polymerase chain reaction (qRT-PCR) to Emr1, TH, and *β*-actin were performed; Immunohistochemistry (IHC) for TH was performed; Enzyme-linked immunosorbent assay (ELISA) was performed to measure serum cytokines. Small intestinal S-IgA and Diamine Oxidase (DAO) activity were also examined. Sugar composition analysis of CPS was conducted.	CPS improves the behavioral deficits in pole tests and gait tests. CPS-treated mice exhibited significant improvement in landing time. Treatment with CPS prevented the decrease in the length of stride. It was found that CPS decreases the reduction of tyrosine hydroxylase, dopamine, and its metabolites within the PD model to prevent DA neuron death. In addition, CPS inhibits the inflammatory process in the central and peripheral nervous systems. CPS enhanced the gut immune system by increasing DAO and S-IgA activities within the MPTP-induced PD model.
Zhang et al. (2015)	Arthrospira (Spirulina) platensis* (Cyanophyceae)	The present study aimed to determine whether a polysaccharide obtained from *A. platensis* (PSP) shows protective effects on dopaminergic neurons. A PD model was established through the intraperitoneal injection of MPTP in male C57BL/6 J mice. Mice in the control group received i.p. injection of normal saline, while mice in the experimental groups were injected with MPTP.	The following tests were carried out: Immunohistochemical determination of tyrosine hydroxylase (TH) and dopamine transporter (DAT) in the substantia nigra pars compacta; RT-PCR detection of TH and DAT mRNA expression in the substantia nigra; Biochemical detection of monoamine oxidase B (MAO-B), superoxide dismutase (SOD), and glutathione peroxidase (GSH-Px) in the serum and midbrain.	Findings indicated that the highest dose of PSP effectively protected against the MPTP-induced loss of TH-positive neurons in the substantia nigra. PSP also attenuated the reduction in TH and DAT expression induced by MPTP. Furthermore, PSP attenuated the decrease in dopamine levels and the increase in dopamine metabolism rates in MPTP-treated mice. We also found that PSP protected against the reduction in SOD and GSH-px caused by MPTP.
Zhang et al. (2018)	*Laminaria japonica* (Pheophyceae)	Male Sprague–Dawley rats were randomly divided into seven groups and underwent treatment for a course of 38 days. Group 1 received 0.9% saline for 10 days and was then cotreated with a vehicle solution, dimethyl sulfoxide (DMSO) + sunflower oil, for 4 weeks. Rats in group 2 received fucoidan (140 mg/kg/d) alone once a day. Rats in group 3 received rotenone from day 11 (5 times a week for 4 weeks) and served as a model group. Rats in groups 4–6 received fucoidan at 35, 70, and 140 mg/kg/d, respectively, for 10 days and were then co-treated with rotenone for 4 weeks. Rats in group 7 received rasagiline for 10 days and were then co-treated with rotenone for 4 weeks which served as a positive control. Fucoidan, rasagiline, and saline were given by gavage. Rotenone and the vehicle solution (DMSO + sunflower oil) were given by a subcutaneous injection.	Behavioral tests (catalepsy and open-field test) were performed. Immunohistochemistry and imaging quantification (TH) were carried out. Levels of DA and its metabolites in the striatum were analyzed using high-performance liquid chromatography. Small tissue blocks excised from the midbrain samples were imaged using a transmission electron microscope. The mitochondrial respiratory function was assayed by measuring oxygen consumption rates (OCRs). The concentration of malondialdehyde (MDA), levels of 3-nitrotyrosine (3-NT) and 8-hydroxy-2-deoxyguanosine (8-OHdG) in the ventral midbrain were measured. Immunoblots to peroxisome proliferator-activated receptor gamma coactivator 1-alpha (PGC-1α), nuclear respiratory factor (NRF2), or β-actin were quantified.	The results obtained from a complete set of experiments demonstrate that fucoidan profoundly rescued the mitochondrial respiratory function in rotenone-lesioned PD rats, while fucoidan at the same time substantially alleviated motor impairments and nigrostriatal dopaminergic degeneration in these rats. Pretreatment with fucoidan and rasagiline significantly alleviated the decrease in four locomotor activities. At 140 mg/kg dose, it reversed the increase in the turnover rate of DA in response to rotenone. The three doses mitigated the rotenone-induced decrease of basal respiration, ATP production, and maximal respiration in a dose-dependent manner.
Xing et al. (2023)	*Fucus vesiculosus* (Pheophyceae)	In total, 34-month-old male C57BL/6 mice were separated into 5 groups: control, MPTP, *Fucus vesiculosus Flour* (FvF), MPTP + FvF (10 mg/kg), and MPTP + FvF (40 mg/kg). FvF was administered daily starting 1 day before MPTP injection. Then, both MPTP and FvF were administered together daily for 5 days. FvF was then provided for another 2 days. Mice in the control group were injected with an equal volume of saline.	Behavioral tests such as open-field test, rotarod test, and pole test were performed after the last administration of MPTP to mice. The time required for the mouse to reach the bottom of the floor (T-TLA) and the time required for the mouse to be fully head down (T-turn) were recorded. The midbrain and stratum tissues of whole brains were quickly dissected, and all samples were stored. For neurotransmitter measurement using HPLC, each stratum was used. For immunofluorescence staining neurons, TH was used.	The impaired motor coordination and balance ability were relieved by FvF. Its treatment significantly slowed the loss of TH markers in the SNc region of MPTP-PD mice. The decrease in DA and DOPAC contents in the striatum was mitigated by FvF but had no effect on the level of 5-HT. FvF treatment significantly slowed the loss of TH markers in the substantia nigra pars compacta (SNpc) region of MPTP-PD mice. The decrease in DA and DOPAC contents in the striatum was mitigated by FvF.
Luo et al. (2009).	*Laminaria japonica* (Pheophyceae)	Experimental Parkinsonism was established through intraperitoneal injections of MPTP. Male mice received fucoidan (once per day) for 18 days while MPTP was given on the 11th day 1 h after injection of fucoidan. Saline-injected but otherwise identically treated mice served as the control group. The animals were sacrificed at different times after MPTP injections to observe the effects of fucoidan.	Locomotor activity was assessed in automated activity chambers. The contents of dopamine and its metabolites, DOPAC and HVA, were determined using an HPLC apparatus. TH positive neurons with distinct nuclei were counted in tissues for histological analysis. Cellular proteins were extracted from striatal samples for the Western blot analysis. TH or GADPH antibodies for detection of the level of dopaminergic neuronal terminals and normalization of the loading protein were used. Striatal tissue was prepared as described above and analyzed for 1-methyl-4-phenylpyridinium (MPP+) level using HPLC and UV detection. The substantia nigra was removed, and the samples were then subjected to the measurement of malondialdehyde (MDA), glutathione (GSH), GSH-Px, SOD, catalase, and total antioxidant capability using spectrophotometric methods.	Total movement distance, mean velocity, and mean distance per movement were partially rescued in fucoidan treatment mice. Fucoidan also protected against the depletion of both striatal dopamine and TH-positive neurons in the substantia nigra pars compacta in the MPTP animal model of Parkinson’s disease. Furthermore, administration of fucoidan effectively limited lipid peroxidation and increased the level/activities of tissue non-enzymic (GSH) and enzymic (SOD, GSH-Px, and catalase) antioxidants of the substantia nigra in MPTP mice.

^*^When the article was published, the name of the alga was *Spirulina platensis*; however, the name of this species has been revised, and currently, *Arthrospira platensis* is considered more accurate. To facilitate recognition of the species and with respect to the authors of the cited study (Sun et al., 2023), the name *Arthrospira (Spirulina) platensis* is used here.

## Discussion

Neuroinflammatory and oxidative stress mechanisms are at the root of the pathogenesis of BG neurodegeneration prior to the onset of its conditions (Singh et al., 2019). Excessive levels of reactive oxygen species (ROS) can modulate the inflammatory response by inducing the expression of pro-inflammatory molecules (Singh et al., 2019). Conversely, the inflammatory response generates ROS through multiple pathways by signaling the pro-inflammatory transcription factor NFκβ (Singh and Singh, 2020). It appears that Nrf2 and the subunit of NFκβ P65 constitute a point of intersection between the antioxidant and inflammatory pathways (George et al., 2022). It is important to highlight that inflammatory molecules can cross the blood–brain barrier and move both toward the peripheral and central nervous systems (Jia et al., 2022).

The 1-methyl-4-phenyl-1,2,3,6-tetrahydropyridine (MPTP) mouse model replicates key pathobiochemical features of PD, including oxidative stress, mitochondrial dysfunction, excitotoxicity, inflammation, and apoptosis (Beal, 2021). Oxidative stress, driven by ROS, significantly contributes to PD pathogenesis, with mitochondria being the primary source of ROS. Polysaccharides have emerged as potential therapeutic agents against PD and Alzheimer’s disease (AD), acting through mechanisms involving neuroinflammation, oxidative stress, autophagy, apoptosis, and mitochondrial dysfunction. Similarly, HD is primarily related to oxidative stress and neurotoxicity (Wang et al., 2022). These shared biological processes play pivotal roles in the progression and pathogenesis of neurodegenerative diseases, prompting extensive research to identify therapeutic targets (Pradhan et al., 2020; Sun et al., 2023; Mohd Sairazi and Sirajudeen, 2020).

Polysaccharides, such as fucoidan, can act both directly by scavenging radicals, chelating metal ions, and donating electrons to unstable molecules and indirectly by increasing the expression, protein level, and activity of antioxidant enzymes (Dhahri et al., 2021).

According to Meenakshi et al. (2016), fucoidan from *Turbinaria decurrens* holds promise as a potential therapeutic agent for PD, impacting dopamine (DA) levels and providing antioxidative benefits. In the striatum of PD animals, DA levels and its metabolites were decreased due to dopaminergic neuron impairment. Pretreatment with this fucoidan effectively reversed the abnormally low levels of 3,4-dihydroxyphenylacetic acid (DOPAC), homovanillic acid (HVA), and DA induced by the action of MPTP. Mice demonstrated a reduction in the levels of thiobarbituric acid reactive substance (TBARS), a product of lipid peroxidation, and an increase in glutathione (GSH) levels and glutathione peroxidase (GPx) activity. As a result, these mice exhibited proper muscular coordination and retained their natural activity.

In their research, Zhang et al. (2014) investigated the effects of fucoidan from *Laminaria japonica* on PD by using a 6-hydroxydopamine (6-OHDA) model. This fucoidan significantly mitigated motor dysfunction and attenuated damage to the dopaminergic system both in the striatum and substantia nigra pars compacta induced by 6-OHDA. The loss of striatal tyrosine hydroxylase (TH-ir) immunoreactive fibers and TH-ir neurons in the central nervous system was associated with behavioral dysfunction, elevated levels of protein carbonylation, microglial activation, and increased expression of Nox1. Fuicodan was capable of suppressing oxidative stress and reducing neuronal damage caused by 6-OHDA by limiting the increase in Nox1. It holds promise as a potential therapeutic agent for PD, acting through mitochondrial support and modulation of antioxidant pathways. This polysaccharide is capable of protecting the dopaminergic pathway, which plays a critical role in motor control. Additionally, it ameliorates the behavioral deficits observed in PD animal models.

In animals exposed to rotenone, a toxin associated with PD pathology, another fuicodan from *L. japonica* exerted protective effects by enhancing mitochondrial function (Zhang et al., 2018). It reduces oxidative stress within the ventral midbrain in rotenone-treated rats. The study suggests that the upregulation of two key proteins—peroxisome proliferator-activated receptor-*γ* coactivator 1 alpha (PGC-1α) and nuclear respiratory factor (NRF2)—significantly contributes to the protective effects of *Laminaria* fucoidan. In the fuicodan treatment group, NRF2 exhibits increased expression, which correlates with enhanced antioxidant activity.

Using an MPTP-induced PD mouse model, fucoidan from *Fucus vesiculosus* demonstrated both neuroprotective and anti-PD effects (Xing et al., 2023). Using target fishing technology, the researchers have identified ATP5F1a, a component of mitochondrial complex V, as the primary target of this fuicodan, which plays a crucial role in reducing ROS. This protein mainly regulates adenosine triphosphate (ATP) production and is involved in cellular energy metabolism. Consequently, it is essential to further investigate the impact of polysaccharides on neuronal cell metabolism.

In the third study, researchers investigated the effects of fucoidan from *L. japonica* on MPTP-induced dopaminergic neuron loss (Luo et al., 2009). Notably, this fucoidan contributed to the increase in the levels of GPx, superoxide dismutase (SOD), catalase (CAT), and GSH in the brain homogenate of the substantia nigra. However, this fuicodan did not affect the pharmacokinetics of MPTP, indicating that its protective effect is not the result of an interaction with the neurotoxin. Furthermore, the increase in TH-positive cells, which are associated with dopaminergic neurons, observed in the fucoidan-treated group is correlated with TH protein levels in the substantia nigra. Notably, this increase exceeds the content of DA and DOPAC. One plausible explanation is that dopaminergic terminals are more sensitive to MPTP toxicity, resulting in more severe damage compared to dopaminergic cell bodies.

Research findings indicate that 6-OHDA increased IL-1β transcription in the rat striatum (Souza et al., 2017). However, treatment with a sulfated polysaccharide obtained from the alga *G. cornea* (SA-Gc) resulted in the modulation of the NFκβ/iNOS/NO2 and NO3 pathways (Souza et al., 2017). SA-Gc promoted the downregulation of pro-inflammatory genes (p65, iNOS, and IL-1β) to basal levels and the upregulation of the brain-derived neurotrophic factor (BDNF) gene. Furthermore, it enhanced GSH production in the striatum, hippocampus, and prefrontal cortex, and caused an improvement in motor functions of 6-OHDA exposed animals. The expression of p65 is associated with anti-inflammatory and antioxidant activity. This protein acts as a crosslink between inflammation and redox homeostasis (George et al., 2022).

Rats treated with SA-Gc exhibited improvements in locomotor disturbances and behavioral changes (Souza et al., 2017). Additionally, an apomorphine-induced rotation test, along with an analysis of striatal monoamine levels, revealed that SA-Gc-treated animals showed significant dose-dependent increases in DA and DOPAC contents. This suggests the integrity of dopaminergic neurons. Furthermore, SA-Gc administration led to weight gain in the hemi-parkinsonian rats, possibly indicating a neuroprotective effect on the maintenance of the dopaminergic pathway.

Polysaccharides isolated from microalgae have also been found to act as protective agents against damage to the central nervous system. Examples of these polysaccharides are those obtained from *Chlorella pyrenoidosa* (Chen et al., 2014) and *Spirulina platensis* (Zhang et al., 2015).

The polysaccharide of *C. pyrenoidosa* (CPS) appears to have anti-inflammatory effects and positively impacts the gut health of animals exposed to MPTP (Chen et al., 2014). Mice exposed to MPTP and treated with CPS exhibited a reduction in the levels of pro-inflammatory cytokines and a decrease in the rate of AD renewal in the striatum. Additionally, the effective reversal of the abnormal drop in serum diaminoxidase activity (DAO) and S-IgA levels in the small intestine was observed. For molecules that do not cross the blood–brain barrier, the mechanism that uses the brain-gut axis pathway appears to be very efficient. This is because SP regulates the gut microbiota and its metabolic products, exerting a neuroprotective effect (Wang et al., 2022).

In a study conducted by Zhang et al. (2015), it was observed that the polysaccharides of *Spirulina platensis* (PSP) had a neuroprotective effect on dopaminergic neurons in a mouse model of PD induced by MPTP. Researchers have found that the protective effect of PSP was not directly related to the inhibition of monoamine oxidase type B (MAO-B), as it did not significantly affect MAO-B activity. Instead, other mechanisms appeared to have been involved. Otherwise, PSP increased the activities of SOD and GPx in mice exposed to MPTP. These enzymes play a crucial role in antioxidant defense, suggesting that the PSP mechanism is related to the modulation of endogenous antioxidant tools and the consequent protection of dopaminergic neurons.

In summary, the species *T. decurrens*, *G. cornea*, *L. japonica*, *C. pyrenoidosa*, *S. platensis,* and *F. vesiculosus* showed significant neuroprotective effects against different neurotoxicity inducers (MPTP and 6-OHDA) through various routes of treatment administration, including intraperitoneal and intrastriatal injections, as well as oral administration. Various forms of evaluation have been employed, including behavior studies, Western blotting analysis, immunohistochemistry, flow cytometry analysis, transcriptional analysis, biochemical detection of enzymes, spectrophotometric methods, liquid chromatography, and quantification of neurotransmitters.

The mechanisms of action behind the neuroprotective benefits suggest that they can act on different targets: directly targeting ROS (Dhahri et al., 2021); enhancing GSH levels (Meenakshi et al., 2016); attenuating Nox1 expressing levels (Zhang et al., 2014); upregulating PGC-1α and NRF2 proteins (Zhang et al., 2018); improving mitochondrial respiratory function by ATP5F1a protein interaction (Xing et al., 2023); increasing the levels of GPx (Luo et al., 2009), SOD (Luo et al., 2009), CAT (Luo et al., 2009), and GSH (Meenakshi et al., 2016; Luo et al., 2009); modulating the transcription of NFκβ/iNOS/NO2 and NO3 pathways (Souza et al., 2017); modulating the immune response through the brain–gut axis (Chen et al., 2014); and increasing the activity of antioxidant enzymes such as GPx (Meenakshi et al., 2016; Zhang et al., 2015) and SOD (Zhang et al., 2015).

The limited number of studies analyzed showed a diversity of lesion models and evaluation methods. The possible mechanisms of protection for polysaccharides from algae in neural damage and diseases associated with the basal ganglia are speculative at this stage, necessitating further investigation.

## Conclusion

The outcomes of this review highlight the potential therapeutic significance of algae-derived polysaccharides. These compounds show promise as neuroprotective agents in areas such as the modulation of inflammation and endogenous antioxidant pathways. They also promoted important amelioration in the morphophysiology of the nervous system under stress. Thus, they may hold value for the treatment of neurodegenerative diseases linked to the basal ganglia. Therefore, the search for neuroprotective polysaccharides in seaweed provides opportunities for researchers to evaluate the potential of these molecules as innovative systems for the prevention and treatment of neurodegenerative diseases. These molecules may also contribute to overcoming the limitations of current treatments.

## Data Availability

The original contributions presented in the study are included in the article/supplementary material, further inquiries can be directed to the corresponding author.
